# Online Feature Selection for Robust Classification of the Microbiological Quality of Traditional Vanilla Cream by Means of Multispectral Imaging

**DOI:** 10.3390/s19194071

**Published:** 2019-09-20

**Authors:** Alexandra Lianou, Arianna Mencattini, Alexandro Catini, Corrado Di Natale, George-John E. Nychas, Eugenio Martinelli, Efstathios Z. Panagou

**Affiliations:** 1 Laboratory of Microbiology and Biotechnology of Foods, Department of Food Science and Human Nutrition, Agricultural University of Athens, Iera Odos 75, 11855 Athens, Greece; alianou@aua.gr (A.L.); gjn@aua.gr (G.-J.E.N.); 2 Department of Electronic Engineering, University of Rome for Vergata, via del Politecnico 1, 00133 Roma, Italy; mencattini@ing.uniroma2.it (A.M.); catini@ing.uniroma2.it (A.C.); dinatale@uniroma2.it (C.D.N.)

**Keywords:** multispectral image analysis, on-line feature selection, adaptive classifier, vanilla cream

## Abstract

The performance of an Unsupervised Online feature Selection (UOS) algorithm was investigated for the selection of training features of multispectral images acquired from a dairy product (vanilla cream) stored under isothermal conditions. The selected features were further used as input in a support vector machine (SVM) model with linear kernel for the determination of the microbiological quality of vanilla cream. Model training (n = 65) was based on two batches of cream samples provided directly by the manufacturer and stored at different isothermal conditions (4, 8, 12, and 15 °C), whereas model testing (n = 132) and validation (n = 48) were based on real life conditions by analyzing samples from different retail outlets as well as expired samples from the market. Qualitative analysis was performed for the discrimination of cream samples in two microbiological quality classes based on the values of total viable counts [TVC ≤ 2.0 log CFU/g (fresh samples) and TVC ≥ 6.0 log CFU/g (spoiled samples)]. Results exhibited good performance with an overall accuracy of classification for the two classes of 91.7% for model validation. Further on, the model was extended to include the samples in the TVC range 2–6 log CFU/g, using 1 log step to define the microbiological quality of classes in order to assess the potential of the model to estimate increasing microbial populations. Results demonstrated that high rates of correct classification could be obtained in the range of 2–5 log CFU/g, whereas the percentage of erroneous classification increased in the TVC class (5,6) that was close to the spoilage level of the product. Overall, the results of this study demonstrated that the UOS algorithm in tandem with spectral data acquired from multispectral imaging could be a promising method for real-time assessment of the microbiological quality of vanilla cream samples.

## 1. Introduction

Quality and safety assurance are of paramount importance for the agro-food industry of the 21st century. Currently food quality and safety assessment are ensured through microbiological, physicochemical and sensory analyses. However, the majority of these methods are labor-intensive, time-consuming, destructive, require experienced personnel, and most importantly, provide retrospective information [1]. Thus, several analytical approaches have been proposed for the non-destructive and rapid monitoring and assessment of food quality and safety including sensor arrays (e.g., electronic noses), spectroscopy methods (e.g., vibrational, NMR or mass spectroscopy techniques), as well as imaging technology approaches [2,3,4,5]. With regard to imaging technology approaches, hyperspectral imaging spectroscopy, coupled with image analysis techniques and chemometric analyses, has been extensively investigated in recent studies for the purpose of food quality evaluation and monitoring [6,7,8,9,10]. Meanwhile, multispectral image analysis, although less studied than hyperspectral imaging, has also been proposed as a promising optical sensing technique for the rapid and non-invasive assessment of the quality attributes of fresh produce commodities [11,12] and the microbiological spoilage of meat and poultry [13,14]. Multispectral imaging (MSI) combines spectral and spatial information, with the former corresponding to the visual and short wave near infrared range of the spectrum and being obtained in discrete bands [15,16]. In this way, this technique has the ability of providing information pertinent to the so-called “surface chemistry” of a given food sample [15].

Hyperspectral images are composed of hundreds of wavelengths for each pixel of the object under study resulting in a high data processing load that may increase the complexity of online application. Moreover, a common problem in pattern recognition is the high collinearity and data redundancy among the variables affecting the robustness of classification or predictive models [17,18]. It is thus necessary to employ feature selection as an important step in identifying the small set of features that carries most of the information contained in the samples [19,20,21,22,23,24]. In this way, the exclusion of non-informative variables from the analysis will result in a simpler model with improved discrimination or prediction capacity. Several feature selection processes have been reported in the literature for developing multispectral imaging quality and safety control systems, based on multivariate methods such as Principal Components Analysis (PCA), Partial Least Squares Regression (PLSR), Stepwise Regression (SE), Successive Projection Algorithm (SPA), Genetic Algorithms (GA) [25]. The selection of the most appropriate approach depends on the nature of the problem under investigation, the size of the dataset, and the kind of sensors used. Feature selection is a crucial part of the previous algorithms and may strongly affect the final performance of classification. In this work, the Unsupervised On-line feature Selection (UOS) approach was employed for feature selection introduced by Magna et al. [26]. This approach is based on two different feature selection procedures. The first feature selection step is applied to the training data removing the non-informative features from the initial dataset. The second procedure is applied every time that a class label has to be assigned to a new sample. For each test sample, a decision-making process uses the training templates and the test sample data to further tune the most appropriate new set of features to build a classification model for the specific test sample. 

In this context, the objective of the present study was to assess the performance of UOS on experimental datasets derived from a work aiming at the evaluation of multispectral imaging as means of characterizing the microbiological quality of a traditional dairy product (vanilla cream).

## 2. Materials and Methods

### 2.1. Vanilla Cream Samples

For a detailed description of the experimental procedure the reader should refer to a previously published work [27]. In brief, the cream samples employed in this work were packages (170 g) of commercially prepared product that were obtained: (a) directly from the manufacturer within three days of production (65 samples), (b) from different retail stores at various points of their shelf life (48 samples) and (c) from expired samples in retail outlets that were returned to the manufacturer (132 samples). The samples were subjected to microbiological analysis for the determination of their microbial load in terms of total viable counts (TVC) whereas at the same time multispectral images were acquired according to the procedures described in Section 2.3. Samples from two different production batches obtained directly from the manufacturer were stored at 4, 8, 12, and 15 °C in high-precision incubators (MIR-153 Sanyo Electric Co., Osaka, Japan) for a maximum period of 40 days. At appropriate time intervals during storage, depending on storage temperature, duplicate samples were subjected to the above-mentioned analyses.

### 2.2. Microbiological Analysis

A weighted amount (25 g) of cream sample was transferred aseptically to a sterile stomacher bag (Seward Medical, London, UK) containing 225 mL of sterilized quarter-strength Ringer’s solution (Lab M Limited, Lancashire, UK) and homogenized in a Stomacher device (Lab Blender 400, Seward Medical) for 1 min at room temperature. For the enumeration of total mesophiles (i.e., total viable counts (TVC)), appropriate serial decimal dilutions in Ringer’s solution were surface plated on tryptic glucose yeast agar (Biolife, Milan, Italy) and colonies were counted after incubation of the plates at 30 °C for 48 h. The obtained microbiological data were expressed as log (colony forming units) per gram of cream (log CFU/g).

### 2.3. Image Acquisition and Analysis

**Figure 1:**
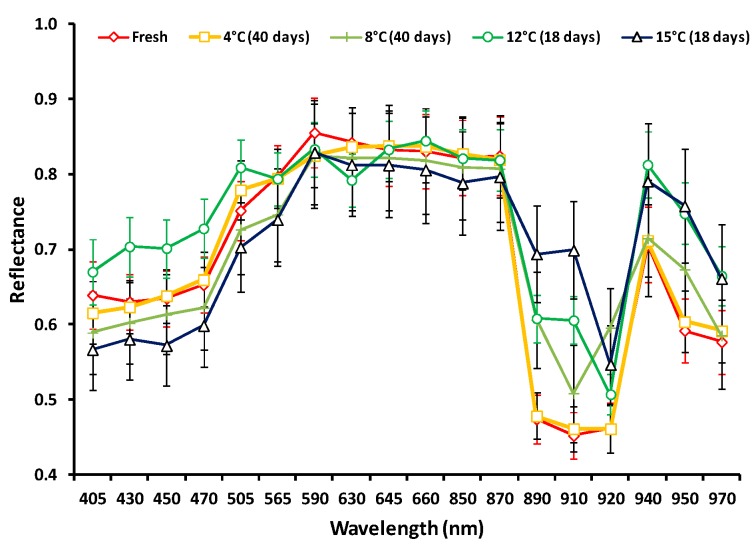
Reflectance spectra (mean ± standard deviation values) of selected vanilla cream samples corresponding to different storage conditions.

Images from each cream sample were acquired using the VideometerLab system originally developed by the Technical University of Denmark [28] and commercialized by “Videometer A/S” (http://www.videometer.com). This instrument acquires multispectral images in 18 different non-uniformly distributed wavelengths ranging from UV (405 nm) to short wave NIR (970 nm). During spectra collection, a data cube of spatial and spectral data of size *m* × *n* × 18 is acquired for each cream sample (*m* × *n* denotes the size in pixels). This process results in the generation of an increased amount of data, representing samples in a time series experiment and/or under different storage conditions (i.e., temperature in this case). Prior to image acquisition the system was subjected to a light set up procedure known as “autolight” and calibrated radiometrically and geometrically as previously described [29]. Each cream sample (i.e., plastic cup after removal of the aluminum lidding foil) was placed inside an Ulbricht sphere in which the camera is top-mounted and the corresponding multispectral image of the product’s surface was acquired. In order to remove redundant information related to the background (e.g., plastic cup) and not to the cream sample *per se*, image segmentation was performed using an in-house method for automated multispectral image analysis. This recently developed method allows for a high-throughput and unbiased analysis of food samples and has been proven to be highly reliable and robust [30]. Briefly, the corresponding processing system embeds a phase of selecting the optimal channel combination without user intervention and, more importantly, regardless of the food sample. Then, the appropriate threshold for the extraction of the informative sample area (i.e., the cream area only) is defined automatically and individually based on Gaussian Mixture Models [31], a robust machine learning technique adapted to this particular application [30]. After the segmentation process of each image, the pixels’ values for each wavelength were normalized into the range [0,1] and the mean reflectance spectrum (i.e., mean intensity of pixels within the informative area) along with the corresponding standard deviation values were calculated. Hence, the multispectral imaging data utilized in subsequent analyses consisted of 18 mean and 18 standard deviation values of the reflectance spectra. A typical spectrum of a fresh vanilla cream sample compared to spoiled samples stored at 4 and 8 °C for 40 days as well as at 12 and 15 °C for 18 days is presented in Figure 1. It is evident that no clear pattern between fresh and spoiled samples could be obtained in the visible region of the spectrum; however, some differences could be established in the near-infrared area, a fact that necessitates the implementation of feature selection for the effective discrimination of the samples in quality classes.

### 2.4. Data Labeling

The overall dataset consisted of 245 spectra of cream samples, from which (a) 65 spectra were acquired during the storage of the samples at isothermal conditions and used in model training, (b) 48 spectra were captured from samples coming from retail outlets at various time points of shelf life and used in model validation, and finally (c) 132 spectra were collected from expired samples and used in model testing. The input matrix for analysis contained as variables (features) the 18 average and 18 standard deviation values of the reflectance spectra acquired from 245 vanilla cream samples, whereas the output matrix contained the results of the microbiological analyses for the determination of TVCs in the respective samples. As a preliminary approach, we intended to apply the procedure to a binary classification problem that consisted in automatically determining the samples having a TVC higher than 6 log CFU/g or smaller than 2 log CFU/g. The idea was to construct a model able to accurately recognize fresh (TVC ≤ 2) or spoiled samples (TVC ≥ 6). The latter threshold value could be considered as the general microbiological guideline applied in food quality [32]. It must be underlined that the value of TVC ≤ 2 is a critical in-house threshold for the Quality Control Department of the dairy industry that supplied the cream samples, because if the population of TVC is less than 2 log CFU/g then the product is released immediately to the market with a designated shelf life of 30 days. So far, the determination of this threshold is defined by expensive, laborious, and time-consuming microbiological analysis. Thus, multispectral imaging could become a low cost, rapid and fully automated method to facilitate the decision-taking process of quality managers in order to release the product in the market, by correlating the outcome of microbiological analysis to multispectral images. As a second step, the recognition model will be applied to those test samples in the range of TVC ∈ (2,6) to assess the capacity of the system to recognize progressively increasing microbial populations. The data labelling scenario is presented in Table 1.

### 2.5. Dynamic Feature Selection (DFS) Method

**Figure 2:**
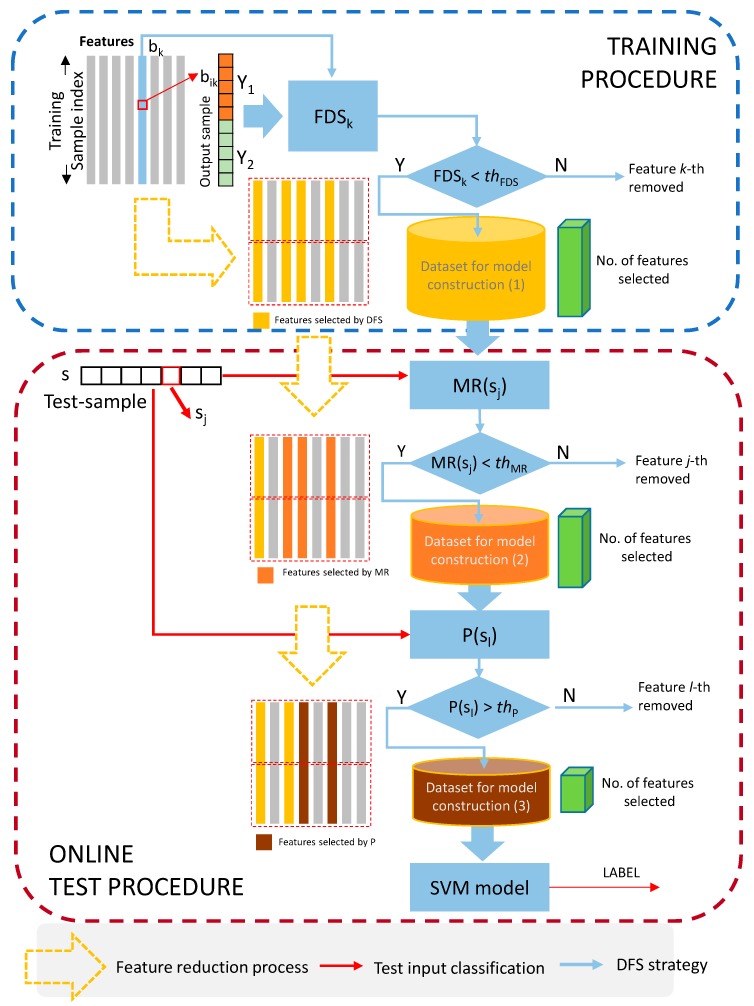
A schematic representation of the Dynamic Feature Selection (DFS) process.

Under the assumption that feature selection plays a crucial role in the recognition performance, especially due to the heterogeneity of the test set, a UOS based on a dynamic feature selection (DFS) procedure intended to optimally select model features according to each specific test data was employed in this work. More specifically, in line with the UOS approach recently developed [26,33,34] the following three-level DFS approach was designed, as summarized in Figure 2.

#### 2.5.1. Training-Dependent Feature Elimination Step

The Fisher Discriminant Score (FDS) is used to sort all the available features in the training set according to their correlation with the classification problem [26]. Considering a two-class problem, let us assume that each class has *L_d_*, d = {1,2} training vectors ${ℬ}_{i}$ each composed by elements $b_{ik}$, *i* = 1, …, *L_d_*, *k* =1, …, *M*, with *M* the total number of features. 

The *FDS_k_* for each feature *k*, is then defined as the ratio between intra-class and inter-class variance and computed as follows:

(1) $$FDS_{k}=SB_{k}/SW_{k}\;,$$

where $SB_{k}$ and $SW_{k}$ are the intra-class and the inter-class variance, respectively. More specifically, $SB_{k}$ is defined as:

(2) $$SB_{k}=\sum_{d=1}^{2}{\left(b_{\left\{Y=Y_{d}\right\}k}-{\bar{b}}_{k}\right)}^{2},$$

with $Y_{d}=\left\{0,1\right\}$, indicating the two class labels, ${\bar{b}}_{k}$ representing the average value of the feature values $b_{k}$ computed over all the classes and *SW_k_* defined as:

(3) $$SW_{k}=\overset{2}{\underset{d=1}{\sum }}\frac{1}{L_{d}}\overset{L_{d}}{\underset{i=1}{\sum }}{\left(b_{{\left\{Y=Y_{d}\right\}}_{i}k}-{\bar{b}}_{dk}\right)}^{2}.$$

with $b_{{\left\{Y=Y_{d}\right\}}_{i}k}$ the *i-th* element of feature *k-th* for the class labelled as $Y_{d}$ and ${\bar{b}}_{dk}$ the average value of *k*-*th* feature in the class labelled as $Y_{d}$. For each feature *k*, $SB_{k}$ quantifies the dispersion of training samples in a class (by their variance) with respect to the global average value of that feature and $SW_{k}$ quantifies the sum of the dispersions of the elements in each class *d* (i.e., the sum of the inter-class variances).

The higher the *FDS_k_* is the more the *k-th* feature is representative of at least one class (high *SB_k_* and low *SW_k_*). On the contrary, features with a *FDS_k_* smaller than a given threshold, here indicated as *th_FD,_* will be immediately rejected (see first decision block in Figure 2). 

#### 2.5.2. Online Test-Dependent Feature Elimination Step

This step utilizes two criteria for further selecting features each time a test set is acquired and processed. The decision is made based on the training set and the test sample newly acquired. This step aims to online remove the features in which either the test sample is surrounded by samples of different classes (high probability of misclassification) or it is far from all class distributions (feature outlier values). For this task, we designed the two following criteria. 

Let us denote with *s* the test sample and with $s_{k}$ its *k*-*th* element, named for brevity test element.

*Determining Feature Outlier Values.* The proposed method is founded on two criteria. Considering the generic feature *k*-*th*, the first criterion is based on the ratio between the Mahalanobis distance of the test sample $s_{k}$ from the distribution of the classes, indicated as *MR_k_* ($s_{k}$). This value is computed as follows:

(4) $$MR_{k}\left(s_{k}\right)=M_{k}\left(s_{k},Y=Y_{1}\right)\cdot \frac{M_{k}\left(s_{k},Y=Y_{1}\right)}{M_{k}\left(s_{k},Y=Y_{2}\right)}\;,$$

where $M_{k}\left(s_{k},Y=Y_{d}\right)$ indicates the Mahalanobis distance of the test sample $s_{k}$ from the training elements belonging to the class labelled as $Y_{d}$ and it is described as:

(5) $$M_{k}\left(s_{k},Y=Y_{d}\right)=\left(s_{k}-{\bar{b}}_{dk}\right)*{\left(Cov\left(b_{\left\{Y=Y_{d}\right\}k}\right)\right)}^{-1}*{\left(s_{k}-{\bar{b}}_{dk}\right)}^{⊺},$$

where $Cov\left(b_{\left\{Y=Y_{d}\right\}k}\right)$ is the sample covariance matrix of the class labelled as $Y_{d}$. The $MR_{k}\left(s_{k}\right)$ gives a quantitative evaluation of the distance of the two classes distribution and the test sample. Lower values of the $MR_{k}\left(s_{k}\right)$ descriptor indicate that the test sample $s_{k}$ is near to a specific class and far from the other one. Therefore, this means that specific feature is representative of at least one class. Hence, for the task of analysing features with a smaller value of $MR_{k}$, we set an additional threshold value *th_MR_* (see second decision block in Figure 2).

*Determining probability of misclassification.* The second criterion is applied during the online feature selection procedure. In this step, the algorithm evaluates the maximum probability *P_k_* for the test example $s_{k}$ to fit to different class distribution. For the *k*-*th* feature, *P_k_* is calculated in the following way:

(6) $$P_{k}\left(s_{k}\right)=\underset{d}{\max}\left(\frac{1}{\sqrt{2\pi }\sigma_{dk}}\exp\left(-\frac{{\left(s_{k}-{\bar{b}}_{dk}\right)}^{2}}{2\sigma_{dk}^{2}}\right)\right),$$

where $\sigma_{dk}$ is the standard deviation of training sample values for feature *k*-*th* in the class labelled as $Y_{d}$. The higher $P_{k}\left(s_{k}\right)$ values are the higher is the probability of the test sample $s_{k}$ to belong to a given class distribution. To evaluate the features that have a higher probability to belong to a given class, we defined a threshold value *th_P_* (see third decision block in Figure 2).

For each test sample s, only the features in the training set that satisfy all the three conditions are considered to build the classification model. 

Among the possible models, in this work we have considered as classifier a Support Vector Machine (SVM) with linear kernel [35]. A new set of features is selected at each new test sample step; it is important to note that features not considered in the online feature selection procedure are re-inserted in the training set and then considered for the feature selection of new test data. 

## 3. Results

### 3.1. DFS Optimization Procedure

**Figure 3:**
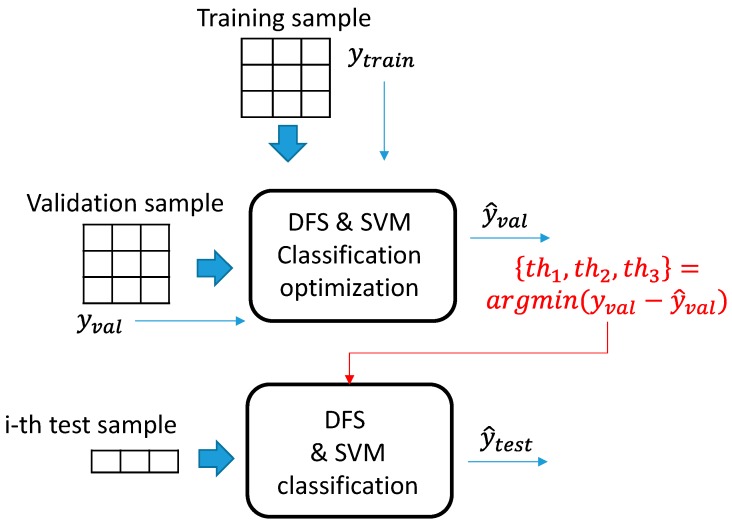
A schematic representation of the procedure for DFS assessment over the MSI dataset.

With the aim to demonstrate the effectiveness of the DFS approach and its superiority with respect to standard feature selection for classification, the assessment procedure shown in Figure 3 was designed. By an exhaustive optimum search procedure, we designed three nested loop procedures in which each threshold, *th*_1_, *th*_2_, *th*_3_ varies within a specific range, *th*_1_ ∈ [0.13–0.17], step size equal to 0.01, *th*_2_ ∈ [0.74–0.77], step size 0.01, *th*_3_ ∈ [0.08–0.12], step size 0.01. At each iteration, the training set along with the corresponding label *y_train_* were fed as training data to the DFS and to the SVM classification model. The SVM model constructed is tested on the validation set thus producing an estimated label vector $\hat{y}$*_val_*. The value is compared against the actual validation label vector y*_val_.* The three thresholds are optimized by minimizing the absolute error between y*_val_* and $\hat{y}$*_val_*. After the thresholds have been set to optimal values, the DFS is run again over the training and test set, the SVM model is constructed over the selected features and an output label is assigned to the test set, $\hat{y}$*_test_*.

### 3.2. Simulation Results

**Figure 4:**
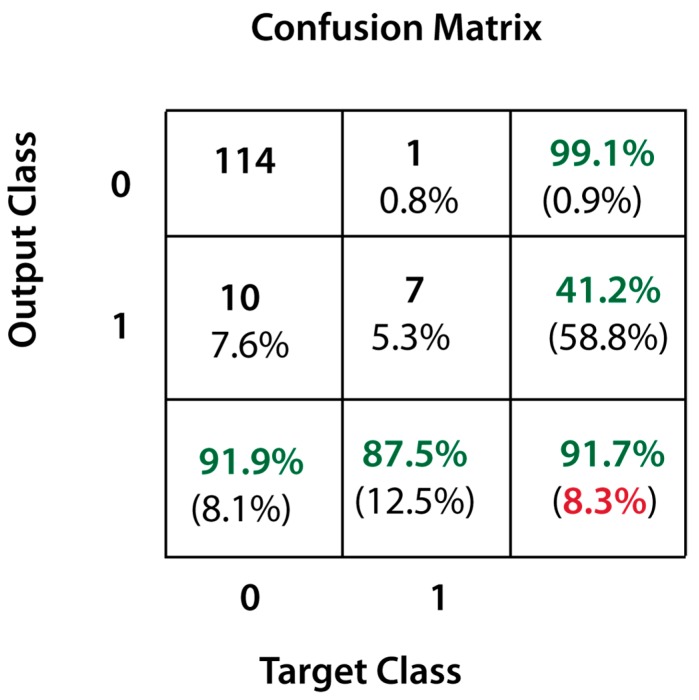
Confusion matrix of the classification results achieved in the fresh (first column) and spoiled (second column) vanilla cream samples.

In a first simulation, we collected the results of the proposed approach obtained when applied over test samples having a TVC smaller than 2 log CFU/g or larger than 6 log CFU/g. Results are presented in terms of accuracy of classification, i.e., the number of total samples correctly classified as fresh or spoiled with respect to the total number of samples in the fresh and spoiled range. Results are shown in terms of confusion matrix in Figure 4. The first column indicates the total number of negative instances in the test dataset (124 fresh samples) and the second column represents the total number of positive instances (8 spoiled samples). The confusion matrix indicates that out of the 124 fresh samples, only 114 were attributed to the negative class (i.e., correctly classified as fresh) and 10 were assigned to the spoiled class (i.e., misclassified as spoiled), leading to a specificity value (true negative rate) of 91.9%. Analogously, out of the eight spoiled samples, seven were assigned to the positive class (i.e., correctly classified as spoiled) and only 1 to the negative class (i.e., misclassified as fresh), resulting in a sensitivity value (true positive rate) of 87.5%. Globally, the system achieved an accuracy value equal to 91.7% for both classes. When applying the same approach also to all the samples in the test dataset we obtained the results reported in Table 2. As a general trend, it can be observed that positive assignments increase with TVC values, whereas negative assignments decrease for TVC increase. Specifically, apart from the fresh (TVC ≤ 2.0 log CFU/g) and spoiled (TVC ≥ 6.0 log CFU/g) samples, the system was able to predict accurately the population of TVC in the range between 2 and 5 log CFU/g. However, as the sample approaches the spoilage condition the percentage of erroneous predictions increases. Reasonably, the most difficult cases are those close to spoilage level. It could be thus suggested that TVC values below 5.0 log CFU/g could be associated with fresh samples, whereas the values of TVC ∈ [5,6) could be regarded as a transition zone to spoilage and could be thus associated to the state of incipient spoilage of vanilla cream samples.

### 3.3. Comparative Analysis

In order to demonstrate the effectiveness of the DFS approach in discriminating fresh from spoiled samples, some additional simulations were performed. Specifically, we decided to compare the following alternative methods: first, we applied different classification algorithms such as Linear and Quadratic Discriminant Analysis (LDA) and (QDA) approaches [36]. Then, we applied a standard Stepwise Regression (SE) static feature selection within a SVM classifier. Results are all presented in Table 3. It can be noted that LDA and QDA did not reach a high accuracy value, while presenting an adequate balancing of the results in critical categories of spoilage (third and fourth columns). This is due to the fact that DFS is the key part of the algorithm and manages to keep high discrimination performance. On the contrary, when DFS was replaced with standard SE feature selection approach, the performance severely got worse at the expense of low accuracy values (70%). More specifically, out of the 124 fresh samples, 74 were assigned to the spoiled category.

## 4. Discussion

Multispectral imaging has been increasingly applied as a powerful analytical tool for rapid and non-destructive determination of food quality and safety, including meat [37,38,39,40], fish [41,42,43], constituent analysis [44,45], adulteration [29,46,47], fruits and vegetables [48,49,50], nuts and grains [51,52] and fungal identification and growth inhibition [53,54]. To our knowledge, there are no published data related to the application and evaluation of multispectral image analysis as a means of appraising the microbiological quality of milk and dairy products. In a recent work [30] a high throughput multispectral image processing pipeline was reported for several foods, including vanilla cream. However, the purpose of that work was not to determine the microbiological quality of the samples during storage, but rather to develop an automated segmentation method for the acquired multispectral images based on unsupervised machine learning approach (Gaussian Mixture Models). An important point in this work is that the developed model included as much variability as possible, as in real life, since model development was based on two different product batches supplied directly by the manufacturer of the vanilla cream that were maintained under laboratory conditions, whereas model testing and validation were based on samples from different retail outlets as well as expired samples from the market. The performance of UOS approach has been successfully used in the past with simulated and experimental datasets for robust classification with drifting and faulty gas sensors [26] as well as in the case of self-repairing classification algorithms for chemical sensor array [55], affective computing [33], and developmental disorders recognition [34]. Not by chance all the considered scenarios present an intrinsic data heterogeneity that makes it usually difficult to develop an effective discrimination strategy using standard paradigms (i.e., diversity of emotion manifesting, autism phenotyping, food spoilage distribution). 

**Figure 5:**
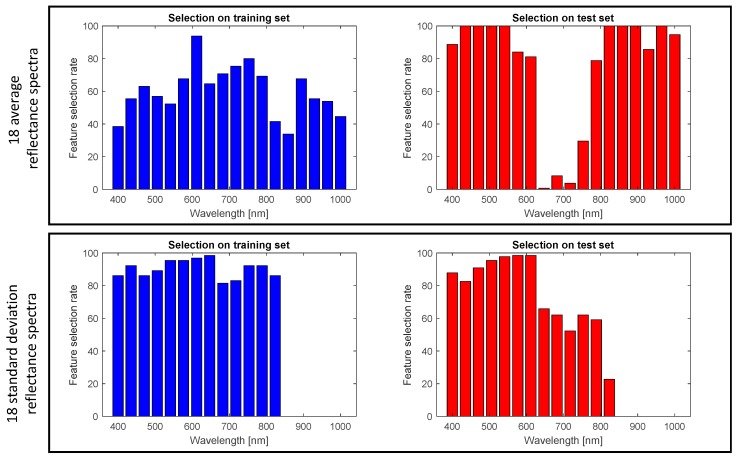
Histogram of the feature selection rate of the 18 average reflectance spectra (**upper**) and the 18 standard deviations of reflectance spectra (**lower**) features for the two datasets. Blue bars refer to the training sample and red bars indicate the test samples. The training results are obtained using a leave-one-out cross validation procedure.

As a further proof of concept, we analyzed how the different features were selected during the online procedure. To this scope, the selected features of the training dataset were firstly analyzed with a leave-one-out cross validation procedure. In a second step, consideration was given on the features selected with the test dataset. The results are shown in Figure 5 where the selection rate for all the features is illustrated. We separated the 18 average reflectance spectra features (upper graph) and the 18 standard deviation reflectance spectra features (lower graph), for the sake of demonstrating different behavior of the two groups of features highlighting the different information content carried by the two kind of descriptors. As it can be observed, there was a strong discrepancy in features selected for different datasets especially for the average reflectance spectra. This demonstrates once more the importance of the DFS approach for such real-life practical scenarios, where unpredictable fluctuations of the experimental conditions can drastically affect the measurement of data. 

It needs to be noted that the UOS processing algorithm was applied for the first time on a food commodity (vanilla cream) with very promising results for the microbiological quality characterization of the product based on the analysis of multispectral images. It is characteristic that 96% of the samples with TVC < 2.0 log CFU/g were correctly classified, a fact that creates new perspectives for the dairy industry to employ multispectral imaging spectroscopy in the quality control pipeline for the rapid estimation of the microbiological quality of vanilla cream and the immediate release of the product to the market.

## 5. Conclusions

The results obtained in this work demonstrated that the UOS algorithm employed for a DFS of spectral data acquired from multispectral images of vanilla cream samples stored at isothermal conditions (4, 8, 12 and 15 °C) for an overall period of 40 days allowed the development of a robust classification model for rapid and non-destructive determination of the microbiological quality of this dairy product for the given set of experimental conditions. The model was extensively tested with independent data from retail outlets at different time points during the shelf-life of the product to include as much variability as possible. As confirmed by the results, the UOS strategy was able to maintain low classification error in the test dataset, especially in the class corresponding to the “fresh” vanilla cream samples, which is used as a criterion by the manufacturer for product release to the market. This fact provides promising perspectives for direct implementation of multispectral imaging in tandem with the UOS strategy in the quality control pipeline of the dairy industry.

## Figures and Tables

**Table 1:** Dataset composition for multispectral imaging (MSI) data. Training and validation samples having TVC counts in the range (2, 6) log CFU/g have been excluded from the analysis. Test dataset is analyzed in total.

Training Data		Validation Data		Test Data		
TVC ≤ 2	TVC ≥ 6	TVC ≤ 2	TVC ≥ 6	TVC ≤ 2	TVC ∈ (2,6)	TVC ≥ 6
29/65	36/65	7/48	41/48	106/132	18/132	8/132

**Table 2:** Classification results for test samples in the whole range of TVC counts obtained during the microbiological analyses of vanilla cream samples.

	Positive Assignment	Negative Assignment
TVC <2	4/103 (4%)	99/103 (96%)
TVC ∈ [2,3)	0/7 (0%)	7/7 (100%)
TVC ∈ [3,4)	0/2 (0%)	2/2 (100%)
TVC ∈ [4,5)	0/2 (0%)	2/2 (100%)
TVC ∈ [5,6)	6/10 (60%)	4/10 (40%)
TVC ≥ 6	7/8 (87.5%)	1/8 (12.5%)

**Table 3:** Classification results of the comparative analysis. Linear Discriminant analysis (LDA) and Quadratic Discriminant Analysis (QDA) are used instead of SVM with the DFS approach. SVM classifier is then used with standard SE feature selection approach. Accuracy values are listed together with a broad report of the sensitivity values for each subcategory of TVC (2,5) and (5,6).

Comparative Analysis			
Method	Accuracy	Sensitivity Per Subcategory	
	TVC < 6 vs. TVC ≥ 6	TVC ∈ (2 ÷ 5)	TVC ∈ (5 ÷ 6)
SVM + DFS	91.7%	0% to SPOILED	60% to SPOILED
LDA + DFS	84.9%	57% to SPOILED	70% to SPOILED
QDA + DFS	85.7%	14% to SPOILED	80% to SPOILED
SVM + SE	70.2%	40% to SPOILED	80% to SPOILED
